# Enhancement and Comparison of (Ceftazidime-)Avibactam Plus Aztreonam Susceptibility Tests for *Stenotrophomonas maltophilia* in Clinical Diagnostics

**DOI:** 10.1007/s00284-023-03530-7

**Published:** 2023-12-05

**Authors:** Gro L. Vlaspolder, Laura A. Hughes, Robert A. G. Huis in ’t Veld, Greetje A. Kampinga, Erik Bathoorn

**Affiliations:** Department of Medical Microbiology and Infection Prevention, University Medical Centre Groningen, University of Groningen, Hanzeplein 1, PO Box 30001, 9700RB Groningen, The Netherlands

## Abstract

*Stenotrophomonas maltophilia* is naturally resistant to many antimicrobials. We evaluated the in vitro activity and reproducibility of two different super-position methods of aztreonam in combination with ceftazidime-avibactam for *S. maltophilia* and compared these results with the recently available aztreonam-avibactam gradient strip. We recommend an improved super-position method that avoids the possible risk of handling a contaminated aztreonam strip. In addition, we report that the cefazidime-avibactam and aztreonam super-position method showed increased in vitro activity in comparison with aztreonam-avibactam indicating activity of the ceftazidime component in vitro.

**Supplementary Information:**

The online version contains supplementary material available at 10.1007/s00284-023-03530-7.

## Introduction

*Stenotrophomonas maltophilia* is intrinsically resistant to many antimicrobials and produces both a metallo-beta-lactamase (MBL) (L1) and a cephalosporinase (L2) [1, 2]. Co-trimoxazole is the first-line treatment [3]. However, resistance to co-trimoxazole can develop, especially in patients with cystic fibrosis who are often colonized with *S. maltophilia* [4].

Several articles have been published regarding the efficacy of the combination of ceftazidime-avibactam (CAZ-AVI) and aztreonam (ATM) against MBL-producing *Enterobacterales*, *Pseudomonas aeruginosa* and *Stenotrophomonas maltophilia* [1, 2, 5, 6]. Avibactam is a beta-lactamase inhibitor with in vitro activity against serine enzymes of the Ambler class A, C and D beta-lactamases, but not against class B MBL.

Avibactam is present at a fixed concentration of 4 mg/L in the CAZ-AVI strips; therefore, it is possible to test the activity of ATM associated with a fixed concentration of AVI using a super-position method. Davido et al. [5] describe a method whereby an ATM gradient strip is applied to Mueller–Hinton (MH) agar for 5 min, and then removed. A CAZ-AVI MIC Test strip (MTS^™^; Liofilchem) is then applied to the same location and then the ATM strip is placed on top of the CAZ-AVI MTS to read the ATM scale. Aztreonam is stable against hydrolysis by MBLs [7]. Consequently, the combination of CAZ-AVI and ATM results in enhanced antimicrobial activity by restoring susceptibility to aztreonam. In this study, we validated and compared methods of super-imposing CAZ-AVI and ATM gradient strips, to determine the in vitro activity against co-trimoxazole-resistant *S. maltophilia* strains.

A disadvantage of this method is the potential spreading of resistant microorganisms when the ATM gradient strip is removed and later replaced. We therefore investigated an alternative super-position method whereby the CAZ-AVI strip is applied to the MH agar for 5 min, then removed and discarded after which an ATM strip is placed in the same location and incubated for 16–20 h, after which the ATM MIC can be read. However, with the recent availability of an ATM-AVI MTS (Liofilchem), the question arises of whether a super-position method to test the susceptibility of *S. maltophilia* to CAZ-AVI and ATM is necessary. Here, we compare the results of *S. maltophilia* susceptibility testing to ATM-AVI MTS with the results of susceptibility testing of *S. maltophilia* to CAZ-AVI and ATM using a super-position method.

### *Stenotrophomonas maltophilia* strains

Thirty-three co-trimoxazole susceptible and resistant *S. maltophilia* strains were included in this study. All isolates were derived from the isolate archive (stored at -80 degrees Celsius) of the Clinical Laboratory for Medical Microbiology within the University Medical Centre Groningen (UMCG) covering a period from 2014 until 2023. The strains were originally cultured from patient material for diagnostic purposes. Each strain was isolated from a unique patient. Since isolates from archived strains were used and no patient data were consulted for this research, no informed consent needed to be obtained.

### Ceftazidime-Avibactam and Aztreonam Super-Position Methods

There are two CAZ-AVI gradient strips available, the Ellipsometry test (Etest^®^; BioMerieux) and the CAZ-AVI MTS. Before comparing the method of Davido et al. [5] and our alternative method with *S. maltophilia* isolates, we first determined the minimum time that a CAZ-AVI gradient strip needed to be in contact with the agar to achieve a consistent MIC using the reference ATCC-strain *K. pneumoniae* ATCC 700603.

We tested the *K. pneumoniae* ATCC 700603 strain in triplicate with the CAZ-AVI Etest and the CAZ-AVI MTS. The strips were placed on the agar and removed after 5 min, 15 min, and 16–20 h of incubation after which the MICs were compared. The MIC values for the CAZ-AVI Etest differed minimally for all time periods tested (supplemental Table S1a). The maximum difference in MICs between the exposure times was a half twofold dilution step. The MIC values for the CAZ-AVI MTS were significantly lower after 16–20 h of incubation compared to 5 and 15 min incubation (supplemental Table S1a)*.* This suggests slower release of antimicrobial from the MTS compared with the Etest.

Based on the finding that placing the Etest on the agar for 5 min was sufficient to achieve a comparable MIC as the reference method, we decided to use this Etest for our alternative super-position method. The alternative super-position method involved placing the CAZ-AVI Etest on the inoculated agar for five minutes at room temperature, after which time the CAZ-AVI Etest was removed and discarded and the ATM Etest was placed on the agar and incubated for a further 16–20 h.

Three *S. maltophilia* strains were used to investigate our alternative method. The isolates were resistant to co-trimoxazole (MIC range >  = 16) and aztreonam (MIC > 256 mg/L). Each *S. maltophilia* isolate was tested in triplicate by three different laboratory technicians using our alternative method and the method described by Davido et al. [5] (supplemental Table S2a) The combination of CAZ-AVI with ATM resulted in a decrease in the aztreonam MIC from > 256 mg/L to 0.25 – 4.0 mg/L for all three isolates. Both methods produced comparable MIC values, which differed by only one twofold dilution step. The MIC values generated by our alternative method differed minimally when tested in triplicate by three technicians (supplemental Table S2a). Our alternative method and the method described by Davido et al. [5] performed comparably for determining the ATM MIC, but the alternative method was more convenient, and likely reduces the risk of cross-contamination.

We then performed our alternative method with 30 other *S. maltophilia* isolates(both co-trimoxazole resistant and co-trimoxazol susceptible). Out of the 33 *S. maltophilia* isolates tested, aztreonam MIC values after CAZ-AVI super-position ranged between 0.38 and 8 mg/L (supplemental Table S2a, Table 1)*.* Of the 33 isolates, 28 had a MIC value in the susceptible category when the EUCAST Pk/Pd breakpoint was applied for ATM (S ≤ 4 mg/L) [8].

**Table 1:** Results of CAZ-AVI and ATM superposition testing compared to ATM-AVI testing for 33 *S. maltophilia* isolates

Isolate	Cotrimoxazole (S/R)*	ATM MIC (mg/L)	CAZ MIC (mg/L)	CAZ-AVI MIC (mg/L)	(ATM 5 min > CAZ-AVI + ATM o/n) MIC ATM (mg/L) **	(CAZ-AVI 5 min > ATM o/n) MIC ATM (mg/L)^***^	ATM-AVI MIC (mg/L)****
1	R	> 256	> *256*	> 256	0.25–0.5	*0.38–0.75*	*1.5–3.0*
2	R	> 256	*1.0*	1.0	0.5–0.75	*0.75–1.0*	*3.0–6.0*
3	R	> 256	> 256	> 256	3.0	3.0–4.0	3.0–8.0
4	R	> 256	*6.0*	1.5	n.t	*0.75*	*3.0–6.0*
5	R	> 256	6.0	1.5	n.t	6.0–8.0	8.0–16
6	R	> 256	> *256*	> 256	n.t	*1.5–2.0*	*4.0–6.0*
7	R	> 256	*4.0*	1.0	n.t	*0.75*	*2.0–3.0*
8	R	> 256	*12*	2.0	n.t	*0.38*	*1.5–2.0*
9	R	> 256	> 256	> 256	n.t	0.75	1.0–1.5
10	R	> 256	> *256*	> 256	n.t	*1.5*	*8.0–12*
11	R	> 256	> 256	32—> 256	n.t	3.0	4.0
12	R	> 256	*8.0–12*	3.0	n.t	*1.5*	*3.0*
13	S	> 256	*4.0*	4.0	n.t	*2.0*	*6.0*
14	S	> 256	*2.0*	0.5	n.t	*1.0*	*4.0*
15	S	64– > 256	> 256	> 256	n.t	2.0	3.0
16	S	> 256	96	96	n.t	6.0	6.0–8.0
17	S	> 256	> 256	> 256	n.t	6.0	6.0
18	R	> 256	> 256	> 256	n.t	4.0–6.0	8.0
19	S	> 256	128	64	n.t	3.0	4.0
20	S	> 256	> 256	> 256	n.t	1.5–2.0	2.0
21	S	> 256	*1.5*	2.0	n.t	*0.5–1.0*	*2.0–3.0*
22	S	> 256	> *256*	> 256	n.t	*2.0*	*4.0*
23	S	> 256	> 256	128	n.t	2.0	1.5–2.0
24	S	> 256	32	6.0	n.t	2.0	3.0
25	R	> 256	> 256	> 256	n.t	4.0	4.0–6.0
26	R	> 256	> 256	> 256	n.t	3.0	3.0
27	R	> 256	> 256	> 256	n.t	6.0–8.0	8.0
28	S	> 256	> 256	> 256	n.t	1.5–2.0	2.0
29	S	> 256	*4.0*	2.0	n.t	*1.5*	*3.0*
30	R	> 256	*48*	32–48	n.t	*4.0*	*8.0*
31	R	> 256	16	12	n.t	2.0–3.0	3.0
32	R	> 256	16–32	24–48	n.t	1.5–2.0	3.0
33	R	> 256	> 256	> 256	n.t	4.0	6.0

Range of MICs determined by Etests or MTS’s. The italics blocks represent the isolates with at least one two-fold dilution difference between the CAZ-AVI + ATM MIC and ATM-AVI MIC

*MIC*,minimal inhibitory concentration, *CAZ-AVI*,ceftazidime-avibactam, *ATM*,aztreonam, *AVI*,avibactam, Etest, Ellipsometry test, *MTS*,MIC Test Strip, *o/n*,overnight incubation, *mg/L* milligrams per litre, *n.t*. not tested, *S*, susceptible, *R* resistant

*Cotrimoxazole susceptibility was determined by Vitek MS, agar diffusion or gradient strip, interpretation of susceptibility was based on EUCAST breakpoints[8]

**Range ATM MIC using the super-position method described by Davido et al. [5], interpreted by three technicians

***Range ATM MIC using the alternative super-position method proposed in this study, starting with incubation of CAZ-AVI test strip for 5 min, followed by ATM overnight incubation; strains 1 to 3 were tested on two different days and were interpreted by three lab technicians; strains 7–10 were tested in duplo and interpreted by one technician. Strains 11–33 were tested singular and interpreted by two technicians

****Range of ATM-AVI MICs; Strains 1–10 were tested in triplicate by three technicians, on two different days, and interpreted by three technicians. Strains 11–33 were tested singular and interpreted by two technicians

### Aztreonam-Avibactam MIC Test Strip

The ATM-AVI MTS was also tested on the *K. pneumoniae* ATCC 700603 strain (supplemental Table S1b) and the 33 *S. maltophilia* isolates (supplemental Table S2b, Table 1). Isolates 1–10 were tested in triplicate by three different laboratory technicians, which was repeated in triplicate on two separate days (Supplemental Table S2b). Isolate 11–33 were tested in *singular*. (Table 1). In isolate 1–10, the ATM-AVI MTS test results showed good repeatability and reproducibility with all values within a single dilution step difference. The results of the *S. maltophilia* strains were compared with the super-position methods (Table 1).

We observed that the CAZ component of the CAZ-AVI and ATM combination appears to have an additional in vitro effect on the susceptibility of *S. maltophilia* isolates when compared to the susceptibility of isolates tested with the ATM-AVI MTS (Table 1). Twenty-four of 33 isolates tested with ATM-AVI showed higher MICs compared to the CAZ-AVI and ATM super-position tests (Table 1).

In 14 isolates, the MICs differed by at least one twofold dilution step (Table 1, strains in italics), and 10 of these 14 isolates, had a lower MIC for CAZ (Table 1). However, slight differences in MICs could possibly be influenced by the differences in material of the gradient strips: the Etests are made of plastic and the MTS are made of paper.

When tested with ATM-AVI MTS, 19 out of 33 isolates tested susceptible for ATM using the cut-off S ≤ 4 mg/L. Whereas only 11 out of 33 isolates tested susceptible applying the Pk/Pd for CAZ-AVI (S ≤ 4 mg/L) compared to 28 out of 33 isolates tested with CAZ-AVI and ATM. Our findings are in line with a recent report investigating mainly co-trimoxazole susceptible *S. maltophilia*, reporting that 96% of isolates are susceptible for CAZ-AVI and ATM, and 16% for CAZ [9]. Future studies may assess which method has a better correlation with treatment outcome: ATM-AVI MTS without the CAZ component, or the CAZ-AVI and ATM super-position method.

In conclusion, we present a method for testing whether the combination of CAZ-AVI and ATM results in the restoration of aztreonam susceptibility in co-trimoxazole resistant *S. maltophilia* isolates with gradient strips that is reproducible and repeatable. The method is more convenient and avoids the possible risk of handling of a contaminated ATM strip compared to the method described by Davido et al*.* The ATM MICs tested lower by the CAZ-AVI and ATM super-position tests compared to the ATM-AVI MTS gradient test, indicating activity of the CAZ component in vitro.

### Supplementary Information

Below is the link to the electronic supplementary material.

Supplementary file1 (DOCX 20 KB)

## Data Availability

The datasets generated and analysed during the current study are available in the supplementary material.

## References

[1] Emeraud C, Escaut L, Boucly A, Fortineau N, Bonnin RA, Naas T (2019). Aztreonam plus clavulanate, tazobactam, or avibactam for treatment of infections caused by metallo-beta-lactamase-producing gram-negative bacteria. Antimicrob Agents Chemother.

[2] Marshall S, Hujer AM, Rojas LJ, Papp-Wallace KM, Humphries RM, Spellberg B (2017). Can ceftazidime-avibactam and aztreonam overcome beta-lactam resistance conferred by metallo-beta-lactamases in enterobacteriaceae?. Antimicrob Agents Chemother.

[3] Falagas ME, Valkimadi PE, Huang YT, Matthaiou DK, Hsueh PR (2008). Therapeutic options for *Stenotrophomonas maltophilia* infections beyond co-trimoxazole: a systematic review. J Antimicrob Chemother.

[4] Esposito A, Pompilio A, Bettua C, Crocetta V, Giacobazzi E, Fiscarelli E (2017). Evolution of *Stenotrophomonas maltophilia* in cystic fibrosis lung over chronic infection: a genomic and phenotypic population study. Front Microbiol.

[5] Davido B, Fellous L, Lawrence C, Maxime V, Rottman M, Dinh A (2017). Ceftazidime-avibactam and aztreonam, an interesting strategy to overcome beta-lactam resistance conferred by metallo-beta-lactamases in enterobacteriaceae and *Pseudomonas aeruginosa*. Antimicrob Agents Chemother.

[6] Mojica MF, Ouellette CP, Leber A, Becknell MB, Ardura MI, Perez F (2016). Successful treatment of bloodstream infection due to metallo-beta-lactamase-producing *Stenotrophomonas maltophilia* in a renal transplant patient. Antimicrob Agents Chemother.

[7] Calvopina K, Hinchliffe P, Brem J, Heesom KJ, Johnson S, Cain R (2017). Structural/mechanistic insights into the efficacy of nonclassical beta-lactamase inhibitors against extensively drug resistant *Stenotrophomonas maltophilia* clinical isolates. Mol Microbiol.

[8] European Committee on Antimicrobial Susceptibility Testing (EUCAST). 2022. Breakpoint tables for interpretation of MICs and zone diameters Version 12.0, valid from 2022–01–01. https://www.eucast.org/fileadmin/src/media/PDFs/EUCAST_files/Breakpoint_tables/v_12.0_Breakpoint_Tables.pdf. Retrieved 7th November 2022.

[9] Mojica MF, Rutter JD, Taracila M, Abriata LA, Fouts DE, Papp-Wallace KM (2019). Population structure, molecular epidemiology, and beta-lactamase diversity among *Stenotrophomonas maltophilia* isolates in the United States. MBio.

