# Supplementary material for: Enhancement and Comparison of (Ceftazidime-)Avibactam Plus Aztreonam Susceptibility Tests for Stenotrophomonas maltophilia in Clinical Diagnostics
Source: Curr Microbiol. 2023 Dec 5;81(1):28. doi: 10.1007/s00284-023-03530-7 (PMC10697871; doi:10.1007/s00284-023-03530-7)
Supplement: Supplementary file 1 — Supplementary file1 (DOCX 20 KB) [file 284_2023_3530_MOESM1_ESM.docx]

**Supplemental Table S1a.** MIC values for *K. pneumoniae* ATCC 700603 determined with Etests and MTS’s when removed after 5 minutes, 15 minutes and 16-20 hours of incubation.

| *K. pneumoniae* (ATCC 700603) | CAZ-AVI Etest  5 min. | CAZ-AVI Etest  15 min. | CAZ-AVI  Etest  16-20 h | CAZ-AVI MTS  5 min. | CAZ-AVI MTS  15 min. | CAZ-AVI MTS  16-20 h |
| --- | --- | --- | --- | --- | --- | --- |
| MIC (mg/L) | 0.75 | 0.75 | 1.0 | 12 | 3.0 | 0.5 |
|  | 0.75 | 1.0 | 1.0 | 16 | 3.0 | 0.75 |
|  | 1.0 | 1.0 | 1.5 | 16 | 4.0 | 0.75 |

**Supplemental Table S1b.** MIC values for *K. pneumoniae* ATCC 700603 determined with the aztreonam-avibactam MTS (loading: 0.016/4 – 256/4 mg/L),

| *K. pneumoniae* (ATCC 700603) | ATM-AVI MTS  Day 1 | Day 2 |
| --- | --- | --- |
| MIC (mg/L) | 0.25 | 0.19 |
|  | 0.19 | 0.19 |
|  | 0.25 | 0.19 |

Abbreviations Table S1a & S1b: CAZ-AVI, ceftazidime-avibactam; ATM-AVI: aztreonam-avibactam; h: hours; Etest, Ellipsometry test; MTS, MIC Test Strip; MIC, minimal inhibitory concentration; mg/L, milligrams per litre; min, minutes.

**Supplemental Table S2a.** All measured results of CAZ-AVI and ATM superposition testing with Etests on ten co-trimoxazole resistant *S. maltophilia* isolates

| Isolate | MIC (mg/L) | | | ATM 5 min > CAZ-AVI o/n*  MIC ATM (mg/L) | | | CAZ-AVI 5 min > ATM o/n**  MIC ATM (mg/L) | | |
| --- | --- | --- | --- | --- | --- | --- | --- | --- | --- |
|  | ATM | CAZ | CAZ-AVI | LT1 | LT2 | LT3 | LT1 | LT2 | LT3 |
| 1 | >256 | >256 | >256 | 0.5 | 0.25 | 0.5 | 0.75 | 0.38 | 0.5 |
| 1# | - | - | - | - | - | - | 0.5; 0.5; 0.5 | 0.5; 0.5; 0.5 | - |
| 2 | >256 | 1.0 | 1.0 | 0.75 | 0.5 | 0.75 | 0.75 | 0.75 | 0.75 |
| 2# | - | - | - | - | - | - | 1.0; 1.0; 10 | 0.75; 1.0; 1.0 | - |
| 3 | >256 | >256 | >256 | 3.0 | 3.0 | 3.0 | 4.0 | 3.0 | 3.0 |
| 3# | - | - | - | - | - | - | 3.0; 3.0; 4.0 | 3.0; 4.0; 4.0 | - |
| 4 | >256 | 6.0 | 1.5 | - | - | - | 0.75; 0.75 | - | - |
| 5 | >256 | 6.0 | 1.5 | - | - | - | 8.0; 6.0 | - | - |
| 6 | >256 | >256 | >256 | - | - | - | 2.0; 1.5 | - | - |
| 7 | >256 | 4.0 | 1.0 | - | - | - | 0.75; 0.75 | - | - |
| 8 | >256 | 12 | 2.0 | - | - | - | 0.38; 0.38 | - | - |
| 9 | >256 | >256 | >256 | - | - | - | 0.75; 0.75 | - | - |
| 10 | >256 | >256 | >256 | - | - | - | 1.5; 1.5 | - | - |

*ATM MIC using the super-position method described by Davido et al. [5], interpreted by three technicians.

** ATM MIC determined by the alternative super-position method proposed in this study, starting with incubation of CAZ-AVI for 5 minutes, followed by ATM overnight incubation; strains 1 to 3 were tested on two different days, interpreted by three technicians; strains 7 to 10 were tested in duplo by one technician.

# Re-test of the isolate by three lab technicians in triplo.

**Supplemental Table S2b.** Results with aztreonam-avibactam MIC Test Strips performed on ten co-trimoxazole resistant *S. maltophilia* isolates

| Isolate | ATM-AVI MTS Day 1  MIC ATM-AVI (mg/L) | | | ATM-AVI MTS Day 2  MIC ATM-AVI (mg/L) | | |
| --- | --- | --- | --- | --- | --- | --- |
|  | LT1 | LT2 | LT3 | LT1 | LT2 | LT3 |
| 1 | 2.0 | 1.5 | 2.0 | 1.5 | 2.0 | 3.0 |
| 2 | 4.0 | 4.0 | 6.0 | 4.0 | 4.0 | 3.0 |
| 3 | 3.0 | 4.0 | 4.0 | 6.0 | 8.0 | 6.0 |
| 4 | 4.0 | 4.0 | 6.0 | 4.0 | 4.0 | 3.0 |
| 5 | 8.0 | 8.0 | 8.0 | 12 | 16 | 12 |
| 6 | 4.0 | 4.0 | 4.0 | 6.0 | 6.0 | 6.0 |
| 7 | 2.0 | 3.0 | 3.0 | 3.0 | 3.0 | 3.0 |
| 8 | 2.0 | 2.0 | 2.0 | 1.5 | 2.0 | 1.5 |
| 9 | 1.5 | 1.0 | 1.5 | 1.0 | 1.0 | 1.0 |
| 10 | 8.0 | 8.0 | 8.0 | 12 | 12 | 8.0 |

Abbreviations Table S2a & S2b: CAZ-AVI, ceftazidime-avibactam; ATM, aztreonam; ATM-AVI, aztreonam-avibactam; Etest, Ellipsometry test; MTS, MIC Test Strip; o/n, overnight incubation; LT, lab technician; MIC, minimal inhibitory concentration; mg/L, milligrams per litre; n.t.: not tested.
